# Affordable flow cytometry for enumeration of absolute CD4^+ ^T-lymphocytes to identify subtype C HIV-1 infected adults requiring antiretroviral therapy (ART) and monitoring response to ART in a resource-limited setting

**DOI:** 10.1186/1479-5876-4-33

**Published:** 2006-08-14

**Authors:** Lynn S Zijenah, Gerard Kadzirange, Simon Madzime, Margaret Borok, Chiedza Mudiwa, Ocean Tobaiwa, Mary Mucheche, Simbarashe Rusakaniko, David A Katzenstein

**Affiliations:** 1 Department of Immunology, College of Health Sciences University of Zimbabwe, Harare, Zimbabwe; 2 Chitungwiza Central Hospital, Chitungwiza, Zimbabwe; 3 Department of Obstetrics and Gynaecology, College of Health Sciences University of Zimbabwe, Harare, Zimbabwe; 4 Department of Medicine, College of Health Sciences University of Zimbabwe, Harare, Zimbabwe; 5 Zimbabwe AIDS Prevention Project, Harare, Zimbabwe; 6 Department of Community Medicine, College of Health Sciences University of Zimbabwe, Harare, Zimbabwe; 7 Division of Infectious Diseases and AIDS Research, Stanford University Medical School, Stanford, California, USA

## Abstract

**Background:**

The World Health Organization (WHO)'s "3 × 5 program" has spurred efforts to place 3 million people on combination antiretroviral therapy (ART) for treatment of AIDS in resource-limited countries. Paradoxically, the cost of CD4^+ ^T-lymphocyte count essential for decision-making to commence HIV positive adults on ART as well as for monitoring responses to ART remains unaffordable in most resource-limited countries. Thus, low-cost methods for enumerating CD4^+ ^T-lymphocyte are urgently needed.

**Objective:**

To evaluate Cyflow cytometry (Cyflow SL, Partec, Munster, Germany) for enumeration of absolute CD4^+ ^T-lymphocyte in subtype C HIV-1 seropositive subjects using FACSCount (Becton and Dickinson, Immunocytometry Systems, San Jose, CA, USA) as the "predicate method".

**Methods:**

A total of 150 HIV-1 seropositive subjects were included in the evaluation exercise. Fifty-eight specimens were collected from pregnant HIV-1 seropositive women (subtype C drug resistance study). Twenty-seven specimens were collected from women and their spouses with AIDS followed in a Duke ART study to assess the immunologic and virologic responses to generic ART, comprising Stavudine, Lamivudine and Nevirapine (Stalanev, Varichem Labs, Harare, Zimbabwe). Sixty-five specimens were collected from AIDS patients enrolled in an ongoing Kaposi Sarcoma (KS) study to investigate impact of ART on KS progression. Enumeration of CD4^+ ^T-lymphocytes using FACSCount is routinely conducted for all the three studies. The Medical Research Council of Zimbabwe and Medicines Control Authority of Zimbabwe approved the studies. Whole blood was collected in EDTA vacutainer tubes and aliquoted into two tubes (200 μL in each). CD4^+ ^T-lymphocyte counts were enumerated using a Cyflow counter, in the Department of Immunology and a FACSCount in the Department of Obstetrics and Gynaecology within 6 hours of phlebotomy following manufacturers' instructions.

**Results:**

Using linear regression analysis, there was a very strong correlation (R = 0.991) between the overall CD4^+ ^T-lymphocyte counts obtained by FACSCount and those obtained by Cyflow. When data analysis was stratified by study groups, there was a strong correlation between the FACSCount and Cyflow CD4^+ ^T-lymphocyte counts from subjects in the three independent studies; Subtype C resistance (R^2 ^= 0.987), Duke ART (R^2 ^= 0.980) and KS (R^2 ^= 0.994), Table 1.

Using Bland-Altman plots, the overall, absolute CD4^+ ^T lymphocytes obtained by the two methods were in excellent agreement (mean difference 1.21, 95% Confidence Interval {CI): -2.1 to 3.3). For the 0–250 CD4^+ ^T-lymphocytes range, the CD4 counts obtained using FACSCount were also in good agreement with those obtained using Cyflow counter (mean difference = 2.6 cells/μL, 95% CI: -1.1 to 6.3). Similarly, in the 251–500 (mean difference 1.0, cells/μL, 95% CI: -3.7 to 5.6) and the 501–1200 (mean difference = 0.29 cells/μL, 95% CI: -8.1 to 8.7) CD4 T-lymphocytes range, good agreement was observed.

**Conclusion:**

The Cyflow counter is as accurate as the FACSCount in enumerating absolute CD4^+ ^T-lymphocytes in the range 1–1200 cells/μL. Cyflow cytometry is relatively affordable, easy to use technology that is useful not only in identifying HIV seropositive individuals who require ART but also for monitoring immunologic responses to ART.

## Background

The World Health Organization (WHO) "3 × 5 program" has spurred efforts to place 3 million people on combination antiretroviral therapy (ART) for treatment of AIDS in resource-limited countries [1,2]. This has been made possible by reduction in price of proprietary ARVs and the introduction of generic versions of these drugs. Paradoxically, the cost of CD4^+ ^T-lymphocyte count essential for decision-making to commence ART for HIV positive adults and to monitor responses to ART remains unaffordable in most resource-limited countries. Although, some resource-limited countries that cannot access affordable CD4^+ ^T-lymphocyte enumeration may use clinical criteria in accordance with WHO recommendations for commencing HIV seropositive individuals on ART [3], CD4^+ ^T-lymphocyte count remains an important measure of immune response, and provide laboratory criteria for stopping co-trimoxazole prophylaxis.

Laboratory-based WHO recommendations for decisions to commence adult HIV-infected individuals on ART are largely based on absolute CD4^+ ^T-lymphocyte counts. Flow cytometry is the gold standard for accurate and automated measurements of absolute T-lymphocyte subset profiles. Flow cytometric techniques are not only expensive, but require sophisticated equipment and reagents as well as highly trained personnel. Furthermore, in resource-limited countries ready access to technical support and quality assurance programs for flow cytometry are often not readily available. Non-flow cytometric methods for counting absolute CD4^+ ^T-lymphocytes such as the manual magnetic bead Dynabead technique have also been evaluated [4,5] but are also considered expensive, labor intensive and can only accommodate low sample throughput [6].

Current clinical flow cytometric methods use either dual or single platforms for enumeration of absolute T-lymphocyte subpopulations. Dual platforms rely on a combination of flow cytometric percentages and white blood cells (WBC) obtained from haematological analyzers to obtain absolute numbers of T-lymphocytes. On the other hand, single platform cytometers are dedicated flow cytometers with direct absolute counting facility. Previous studies have shown that dual platforms have a higher inter-laboratory percent coefficient of variation (%CV) of absolute CD4^+ ^T-lymphocytes compared to single platforms [7]. The higher %CV was attributed to use of different haematological analyzers, with no external quality assurance and internal quality control for WBC used in dual platforms [8]**and references therein**.] Single platform flow cytometers may allow the efficient and accurate enumeration of T-lymphocyte subpopulations in clinical settings where large numbersof patients are receiving care [9].

Recently, great strides have been made in developing easy to use affordable single platform flow cytometric methods for enumerating absolute CD4^+ ^T-lymphocytes. For example, Janossy and colleagues introduced primary CD4 gating on volumetric flow cytometry for absolute CD4^+ ^T-lymphocyte counts without microbeads or haematology [10]. However, this method was developed using the 'state of the art' flow cytometers (Cytoron Ortho and FACSCalibur) with the attendant capacity and costs requirements.

Recently, an increasing number of point of care less expensive methods to measure absolute CD4^+ ^T-lymphocyte counts have been developed. These include the development and evaluation of simplified single platform flow cytometric methods using a low cost flow cytometer that can be powered from a car battery or by solar panels (Cyflow SL, Partec, Munster, Germany) by Cassens and colleagues [11,12], modification of a commercially available 4-parameter flow cytometer, Luminex 100 (Luminex, Austin Texas, USA) to a compact portable prototype instrument that can operate with a 12-volt rechargeable battery [13] and Guava PCA System (Guava Technologies, Hayward, CA, USA).

The objective of the current study was to evaluate single platform Cyflow cytometry (Cyflow SL, Partec, Munster, Germany) for enumeration of absolute CD4^+ ^T-lymphocytes among subtype C HIV-1 seropositive subjects using single platform FACSCount (Becton and Dickinson, Immunocytometry Systems, San Jose, CA, USA) as the predicate method.

## Methods

A total of 150 HIV-1 seropositive subjects were included in the evaluation exercise. Fifty-eight specimens were collected from pregnant HIV-1 seropositive women (subtype C drug resistance study). Twenty-seven specimens were collected from women and their spouses with AIDS followed in a study to assess the immunologic and virologic responses to generic ART (Stalanev, Varichem Labs, Harare, Zimbabwe) comprising Stavudine, Lamivudine and Nevirapine. Sixty-five specimens were collected from AIDS patients enrolled in an ongoing Kaposi Sarcoma (KS) study to investigate impact of ART on KS progression. Enumeration of CD4^+ ^T-lymphocytes using FACSCount is routinely conducted for all the three studies. In the current evaluation of the Cyflow Counter, the FACS counter is thus used as the "predicate method". The Medical Research Council of Zimbabwe and Medicines Control Authority of Zimbabwe approved the studies.

Whole blood was collected in EDTA vacutainer tubes (Becton-Dickinson, Mt View, California) and aliquoted into two tubes (200 μL in each). One tube was sent to the Department of Immunology and the second to the Department of Obstetrics and Gynaecology for enumeration of CD4^+ ^T-lymphocytes using a Cyflow counter and a FACSCount respectively within 6 hours of phlebotomy following manufacturers' instructions. The two independent medical laboratory scientists in the two Departments who conducted the enumeration of the CD4^+ ^T-lymphocytes were blinded to each other's results.

Cyflow reagents and consumables were used according to the manufacturer's instructions. Briefly, 20 μL of whole EDTA blood was pipetted into a Partec test tube. Ten microlitres of CD4-phycoeryrthrin conjugated monoclonal antibody supplied by Partec was added to the tube containing whole blood and the reactants incubated for 15 minutes at room temperature. Following incubation, 800 μL of no lyse buffer, supplied by Partec were added to the tube and gently vortex-mixed. The tube was then plugged into the Cyflow counter for automatic counting. The histogram and absolute counts are displayed and printed automatically. The histogram shows direct counting result in terms of absolute CD4^+ ^T-lymphocytes/μL. The CD4^+ ^T-lymphocytes with high fluorescence appear in a prominent peak at the right of the histogram, whereas the weaker but also CD4^+ ^monocytes appear to the left without any overlap with CD4^+ ^T-lymphocytes.

For FACSCount analysis, 50 μL of whole EDTA blood was added to the CD4 FACSCount reagent tube containing anti-CD3 and anti-CD4 monoclonal antibodies, sample diluent, and reference beads. The samples were then incubated at room temperature for 60 minutes and subsequently run on the FACSCount.

### Statistical analysis

Correlation coefficient and linear regression analysis were used to compare results obtained by FACSCount and Cyflow. CD4^+ ^T-lymphocytes counts obtained by FACSCount were plotted on the X-axis against counts obtained by Cyflow counter on the Y-axis. Bland-Altman plots [14] were used to test the agreement between the two methods by plotting the mean of the values obtained by the two methods on the X-axis and the difference between the two methods on the Y-axis. The mean difference between the two methods (bias) and its 95% confidence intervals, and the limits of agreement, defined as bias ± 2 standard deviation were calculated. Sub-analysis of data was also performed separately for the three independent studies from which subjects were recruited. Data analysis was also stratified according to the range (0–250; 251–500 and 501–1200 cells/μL) of absolute CD4^+ ^T-lymphocyte counts to determine any range-dependent differences between the two methods.

## Results

A total of 150 specimens from HIV seropositive patients with CD4^+ ^T-lymphocyte counts ranging from 1 to 1200 cells/μL were evaluated. The overall median CD4^+ ^T-lymphocyte count of the cohort using the FACSCount was 371 cells/μL (Interquartile range {IQR: 240–513), whilst that from the Cyflow counter was 362 cells/μL {IQR: 244–530).

**Figure 1:**
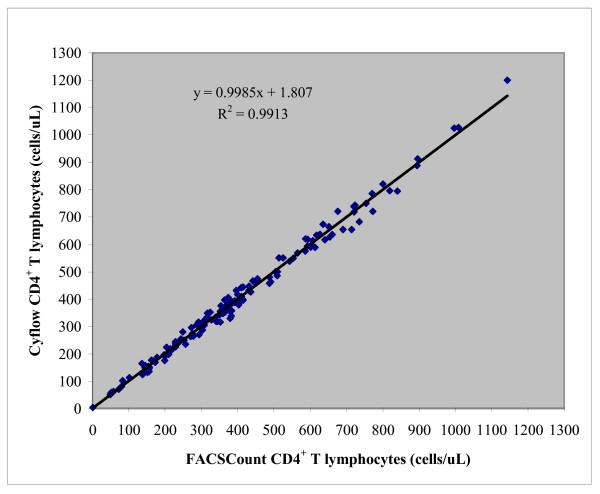
Correlation and R^2 ^values for CD4^+ ^T-lymphocytes obtained by Cyflow and FACSCount.

Linear regression plot of absolute CD4^+ ^T-lymphocytes obtained by Cyflow and FACSCount is shown in Figure 1. Correlation analysis of absolute CD4^+ ^T-lymphocytes obtained by the two methods is shown in Table 1. There was a very strong correlation (R = 0.995, R^2 ^= 0.991) between the overall CD4^+ ^T-lymphocyte counts obtained by FACSCount and that obtained by Cyflow counter (Table 1).

**Table 1:** Correlation analysis of CD4^+^T-lymphocyte counts obtained by FACSCount and Cyflow

**Study group**	**Mean difference CD4**	**SD**	**R^2^**	**y**	**Intercept**	**95% CI**
All (N = 150)	1.21	20.40	0.991	0.998x + 1.807	1.807	-5.09 to 8.70
Subtype C drug resistance (N = 58)	2.40	18.11	0.987	1.0009x + 1.951	1.951	-15.46 to 19.36
Duke ART (N = 27)	-2.04	18.88	0.980	0.9628x + 15.708	15.708	-6.51 to 37.92
Kaposi Sarcoma ART (N = 65)	-0.18	15.77	0.994	0.9949x + 1.432	1.432	-5.83 to 8.70

**Range of CD4^+ ^T-lymphocytes**	**Mean difference CD4**	**SD**	**R^2^**	**y**	**Intercept**	**95% CI**

0–250 CD4^+ ^cells/μL (N = 40)	2.60	11.94	0.967	0.9653x + 3.022	3.0216	-7.25 to 13.30
251–500 CD4^+ ^cells/μL (N = 69)	0.96	19.59	0.898	0.9275x + 25.443	25.443	-2.64 to 53.53
501–1200 CD4^+ ^cells/μL (N = 41)	0.29	27.49	0.968	0.9456x + 37.090	37.090	-2.04 to 76.24

When data analysis was stratified by study groups, there was a strong correlation between the FACSCount and Cyflow counter CD4^+ ^T-lymphocyte counts from subjects in the three independent studies; Subtype C resistance (R = 0.993, R^2 ^= 0.987), Duke ART (R = 0.990, R^2 ^= 0.980) and KS (R = 0.997, R^2 ^= 0.994), Table 1. Similarly strong correlations were observed when data analysis was stratified by CD4^+ ^T-lymphocytes range; 0–250 (R = 0.983, R^2 ^= 0.966), 251–500 (R = 0.948, R^2 ^= 0.898) and 501–1200 (R = 0.984, R^2 ^= 0.968) cells/μL.

**Figure 2:**
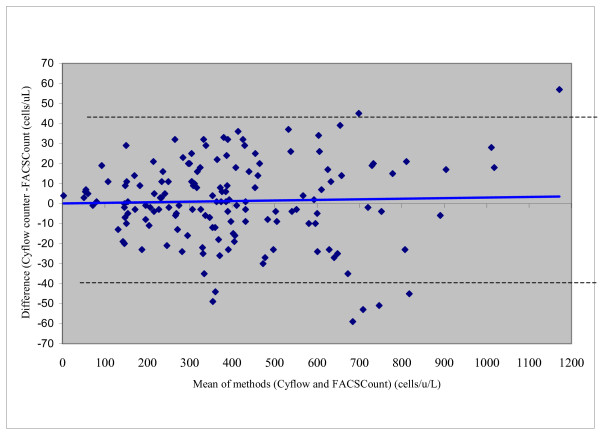
Bland-Altman plot comparing absolute CD4^+ ^T-lymphocyte counts obtained by Cyflow and FACSCount. The blue line indicates the bias (mean difference) and the dotted lines are limits of agreement (mean ± 2 SD). Mean = 1.21 (95% CI: -2.1 to 3.3); limits of agreement between +42 and -40.

Figure 2 shows the Bland-Altman plot comparing CD4^+ ^T-lymphocytes obtained by Cyflow and FACSCount. The overall, absolute CD4^+ ^T lymphocytes obtained by the two methods were in excellent agreement (mean difference = 1.21, 95% Confidence Interval (CI: -2.1 to 4.5). For the 0–250 CD4^+ ^T-lymphocytes range, the CD4 counts obtained using FACSCount were also in good agreement with those obtained using Cyflow counter (mean difference = 2.6 cells/μL, 95% CI: -1.1 to 6.3), Table 2. Similarly, in the 251–500 (mean difference = 0.96, cells/μL, 95% CI:-3.7 to 5.6) and the 501–1200 (mean difference = -0.29, cells/μL, 95% CI: -8.1 to 8.7) CD4^+ ^T-lymphocytes ranges, good agreement was observed (Table 2).

**Table 2:** Bland-Altman analysis comparison of Cyflow versus FACSCount absolute CD4^+ ^T-lymphocyte counts

**Range of CD4^+ ^T-lymphocyte counts**	**N**	**Positive and negative LOA (cells/μL)**	**Mean difference (bias)**	**95% CI of the mean**
0–1200 CD4^+ ^cells/μL (All)	150	42 to -40	1.21	-2.1 to 3.3
0–250 CD4^+ ^cells/μL	40	27 to -21	2.6	-1.1 to 6.3
251–500 CD4^+ ^cells/μL	69	40 to -38	0.96	-3.7 to 5.6
501–1200 CD4^+ ^cells/μL	41	55 to -55	0.29	-8.1 to 8.7

N; Number tested; CI, confidence interval; LOA, limits of agreement

WHO recommends that HIV seropositive adults with CD4^+ ^T-lymphocyte ≤ 200 cells/μL should be commenced on ART [3], we thus evaluated the accuracy of the Cyflow counter in identifying these individuals. The Cyflow counter concordantly identified all but one subject (26/27) with CD4^+ ^T-lymphocyte counts of ≤200 cells/μL as determined by FACSCount. The CD4^+ ^T-lymphocyte counts for the subject with discordant results were 210 and 199 cells/μL from FACSCount and Cyflow respectively.

## Discussion

ARVs have become increasingly available in resource-limited countries in the past few years, mainly because of drastic reductions in prices of proprietary and generic drugs. However, the cost of enumerating absolute CD4^+ ^T-lymphocyte required for decision-making to commence HIV infected individuals on ART and monitoring responses to ART remains unaffordable for most resource-limited countries. Recently, development of affordable practical technologies for enumeration of absolute CD4^+ ^T-lymphocyte may play a critical role in making ART available to all those that need it in developing countries.

In the current study, we have shown that the single platform Partec Cyflow counter can accurately identify HIV seropositive subjects with less than 200 CD4 cells, who are eligible for ART, and conversely, those with > 200 CD4 cells who may not require continued Co-trimoxazole prophylaxis. Furthermore, we have also shown that Partec Cyflow counter can provide accurate CD4^+ ^T-lymphocyte counts for patients who are on ART.

Our results are in agreement with those reported from a recent multicenter evaluation involving eight hospitals in Europe, Africa and Asia [12] as well as independent evaluations in several other resource-limited countries that have evaluated Partec Cyflow counter for enumeration of absolute CD4^+ ^T-lymphocyte counts albeit using various versions of Cyflow counter. In Senegal, where subtypes A and D HIV-1 predominate, an excellent correlation was observed when Partec Cyflow SL blue (equipped with 3 fluorescence and 2 light scatter detectors) was compared to FACScan and FACSCount [15]. In another West African country, Nigeria, with similar co-epidemics, the Cyflow technique was reported to be more precise and cost-effective than the Dynabead method [6]. A study in Malawi, where subtype C predominates, field evaluation of Cyflow reported a correlation coefficient of 0.92 (95% CI 0.89–0.95) when compared to FACSCount with trained technologists finding Cyflow counter procedures simple to run and the instrument easy to manipulate [16]. A good correlation between CD4^+ ^T-lymphocyte counts obtained by Cyflow and FACSCount has also been reported in Thailand where subtypes B HIV-1 predominates and CRF AE_01 [17]. Most importantly, when comparing two methods of clinical measurements, in all these studies, Bland-Altman plots showed good agreement between Cyflow and the "predicate method".

There is an urgent need to develop, evaluate and implement more affordable and easy to use technology for enumerating CD4^+ ^T-lymphocyte counts to commence patients on ART as well as for monitoring immunologic responses to ART to make ART available to all those who need it, particularly in resource-limited countries.

Currently in Zimbabwe, the cost of CD4^+ ^T-lymphocyte count is US$56. This cost is revised upward quarterly. According to the manufacturer of Cyflow, a CD4^+ ^T-lymphocyte count using their machine and reagents should cost only US$2.50 (2 Euros). Although the purchasing price of FACSCount and Cyflow counter are comparable (US$30 000-50 000), the FACSCount reagents are more expensive than those for the Cyflow. The Cyflow counter also has a high throughput and as many as 200 specimens can be run per day, making it ideal for use in Zimbabwe, a country with one of the highest prevalence of HIV globally.

In conclusion, the Cyflow counter is as accurate as the FACSCount in enumerating absolute CD4^+ ^T-lymphocytes in the range 1–1200 cells/μL. Cyflow cytometry is relatively affordable, easy to use technology that is useful not only in identifying HIV seropositive individuals who require ART but also for monitoring immunologic responses to ART.
